# Supplementary material for: Development of a computer-aided design software for the quantitative evaluation of aesthetic damage
Source: PLoS One. 2019 Dec 18;14(12):e0226322. doi: 10.1371/journal.pone.0226322 (PMC6919621; doi:10.1371/journal.pone.0226322)
Supplement: S1 Code site — 1. (ZIP) [file pone.0226322.s001.zip › [EESC_jr][Projeto]Relatorio_07_21/vendors/select2/docs/_includes/examples/tokenizer.html]

# Automatic tokenization

Select2 supports ability to add choices automatically as the user is
typing into the search field. Try typing in the search field below and
entering a space or a comma.

The separators that should be used when tokenizing can be specified
using the tokenSeparators
options.

red
blue
green

{% highlight js linenos %}
$(".js-example-tokenizer").select2({
tags: true,
tokenSeparators: [',', ' ']
})
{% endhighlight %}
